# Systematic review on the frequency and quality of reporting patient and public involvement in patient safety research

**DOI:** 10.1186/s12913-024-11021-z

**Published:** 2024-04-26

**Authors:** Sahar Hammoud, Laith Alsabek, Lisa Rogers, Eilish McAuliffe

**Affiliations:** 1 UCD Centre for Interdisciplinary Research, Education and Innovation in Health Systems (UCD IRIS), School of Nursing, Midwifery and Health Systems, Health Sciences Centre, University College Dublin, Dublin, Ireland; 2 Department of Oral and Maxillofacial Surgery, University Hospital Galway, Galway, Ireland

**Keywords:** Patient and public involvement, Patient participation, PPI, Research reporting, Research involvement, Patient safety

## Abstract

**Background:**

In recent years, patient and public involvement (PPI) in research has significantly increased; however, the reporting of PPI remains poor. The Guidance for Reporting Involvement of Patients and the Public (GRIPP2) was developed to enhance the quality and consistency of PPI reporting. The objective of this systematic review is to identify the frequency and quality of PPI reporting in patient safety (PS) research using the GRIPP2 checklist.

**Methods:**

Searches were performed in Ovid MEDLINE, EMBASE, PsycINFO, and CINAHL from 2018 to December, 2023. Studies on PPI in PS research were included. We included empirical qualitative, quantitative, mixed methods, and case studies. Only articles published in peer-reviewed journals in English were included. The quality of PPI reporting was assessed using the short form of the (GRIPP2-SF) checklist.

**Results:**

A total of 8561 studies were retrieved from database searches, updates, and reference checks, of which 82 met the eligibility criteria and were included in this review. Major PS topics were related to medication safety, general PS, and fall prevention. Patient representatives, advocates, patient advisory groups, patients, service users, and health consumers were the most involved. The main involvement across the studies was in commenting on or developing research materials. Only 6.1% (*n* = 5) of the studies reported PPI as per the GRIPP2 checklist. Regarding the quality of reporting following the GRIPP2-SF criteria, our findings show sub-optimal reporting mainly due to failures in: critically reflecting on PPI in the study; reporting the aim of PPI in the study; and reporting the extent to which PPI influenced the study overall.

**Conclusions:**

Our review shows a low frequency of PPI reporting in PS research using the GRIPP2 checklist. Furthermore, it reveals a sub-optimal quality in PPI reporting following GRIPP2-SF items. Researchers, funders, publishers, and journals need to promote consistent and transparent PPI reporting following internationally developed reporting guidelines such as the GRIPP2. Evidence-based guidelines for reporting PPI should be encouraged and supported as it helps future researchers to plan and report PPI more effectively.

**Trial registration:**

The review protocol is registered with PROSPERO (CRD42023450715).

**Supplementary Information:**

The online version contains supplementary material available at 10.1186/s12913-024-11021-z.

## Background

Patient safety (PS) is defined as “the absence of preventable harm to a patient and reduction of risk of unnecessary harm associated with healthcare to an acceptable minimum” [1]. It is estimated that one in 10 patients are harmed in healthcare settings due to unsafe care, resulting in over three million deaths annually [2]. More than 50% of adverse events are preventable, and half of these events are related to medications [3, 4]. There are various types of adverse events that patients can experience such as medication errors, patient falls, healthcare-associated infections, diagnostic errors, pressure ulcers, unsafe surgical procedures, patient misidentification, and others [1].

Over the last few decades, the approach of PS management has shifted toward actively involving patients and their families in managing PS. This innovative approach has surpassed the traditional model where healthcare providers were the sole managers of PS [5]. Recent research has shown that patients have a vital role in promoting their safety and decreasing the occurrence of adverse events [6]. Hence, there is a growing recognition of patient and family involvement as a promising method to enhance PS [7]. This approach includes involving patients in PS policy development, research, and shared decision making [1].

In the last decade, research involving patients and the public has significantly increased. In the United Kingdom (U.K), the National Institute for Health Research (NIHR) has played a critical role in providing strategic and infrastructure support to integrate Public and Patient Involvement (PPI) throughout publicly funded research [8]. This has established a context where PPI is recognised as an essential element in research [9]. In Ireland, the national government agency responsible for the management and delivery of all public health and social services; the National Health Service Executive (HSE) emphasise the importance of PPI in research and provide guidance for researchers on how to involve patients and public in all parts of the research cycle and knowledge translation process [10]. Similar initiatives are also developing among other European countries, North America, and Australia. However, despite this significant expansion of PPI research, the reporting of PPI in research articles continues to be sub-optimal, inconsistent, and lacks essential information on the context, process, and impact of PPI [9]. To address this problem, the Guidance for Reporting Involvement of Patients and the Public (GRIPP) was developed in 2011 following the EQUATOR methodology to enhance the quality, consistency, and transparency of PPI reporting. Additionally, to provide guidance for researchers, patients, and the public to advance the quality of the international PPI evidence-base [11]. The first GRIPP checklist was a significant start in producing higher-quality PPI reporting; however, it was developed following a systematic review, and did not include any input from the international PPI research community. Given the importance of reaching consensus in generating current reporting guidelines, a second version of the GRIPP checklist (GRIPP2) was developed to tackle this problem by involving the international PPI community in its development [9]. There are two versions of the GRIPP2 checklist, a long form (GRIPP2-LF) for studies with PPI as the primary focus, and a short form (GRIPP2-SF) for studies with PPI as secondary or tertiary focus.

Since the publication of the GRIPP2 checklist, several systematic reviews have been conducted to assess the quality of PPI reporting on various topics. For instance, Bergin et al. in their review to investigate the nature and impact of PPI in cancer research, reported a sub-optimal quality of PPI reporting using the GRIPP2-SF, mainly due to failure to address PPI challenges [12]. Similarly, Owyang et al. in their systematic review to assess the prevalence, extent, and quality of PPI in orthopaedic practice, described a poor PPI reporting following the GRIPP2-SF checklist criteria [13]. While a few systematic reviews have been conducted to assess theories, strategies, types of interventions, and barriers and enablers of PPI in PS [5, 14–16], no previous review has assessed the quality of PPI reporting in PS research. Thus, our systematic review aims to address this knowledge gap. The objective of this review is to identify the frequency PPI reporting in PS research using the GRIPP2 checklist from 2018 (the year after GRIPP2 was published) and the quality of reporting following the GRIPP2-SF. The GRIPP2 checklist was chosen as the benchmark as it is the first international, evidence-based, community consensus informed guideline for the reporting of PPI in research and more specifically in health and social care research [9]. Additionally, it is the most recent report-focused framework and the most recommended by several leading journals [17].

## Methods

We followed the Preferred Reporting Items for Systematic Reviews and Meta-Analyses (PRISMA) guidelines to plan and report this review [18]. The review protocol was published on PROSPERO the International Database of Prospectively Registered Systematic Reviews in August 2023 (CRD42023450715).

### Search strategy

For this review, we used the PICo framework to define the key elements in our research. These included articles on patients and public (P-Population) involvement (I- phenomenon of Interest) in PS (C-context). Details are presented in Table 1. Four databases were searched including Ovid MEDLINE, EMBASE, PsycINFO, and CINAHL to identify papers on PPI in PS research. A systematic search strategy was initially developed using MEDLINE. MeSH terms and keywords relevant to specific categories (e.g., patient safety) were combined using the “OR” Boolean term (i.e. patient safety OR adverse event OR medical error OR surgical error) and categories were then combined using the “AND” Boolean term. (i.e. “patient and public involvement” AND “patient safety”). The search strategy was adapted for the other three databases. Full search strategies are provided in Supplementary file 1. The search was conducted on July 27th, 2023, and was limited to papers published from 2018. As the GRIPP2 tool was published in 2017, this limit ensured the retrieval of relevant studies. An alert system was set on the four databases to receive all new published studies until December 2023, prior to the final analysis. The search was conducted without restrictions on study type, research design, and language. To reduce selection bias, hand searching was carried out on the reference lists of all the eligible articles in the later stages of the review. This was done by the first author. The search strategy was developed by the first author and confirmed by the research team and a Librarian. The database search was conducted by the first author.

**Table 1:** Search terms used in search strategy

P-Population	I- phenomenon of Interest	C-context
Patient	Empower	Patient safety
Public	Involve	Medication error
Community	Engage	Surgical error
Citizen	Participate	Communication error
Family	Consult	Adverse event
Carer	Partner	Medical harm
Caregiver	Collaborate	Medical injury
Relative	Contribute	Missed care
Consumer	Activate	Near miss
User	Co-design	Fall
Client	Co-produce	Slip
Customer		Trip
		Accident prevention
		Patient harm
		Risk management

### Inclusion and exclusion criteria

Studies on PPI in PS research with a focus on health/healthcare were included in this review. We defined PPI as active involvement which is in line with the NIHR INVOLVE definition as “research being carried out ‘with’ or ‘by’ members of the public rather than ‘to’, ‘about’ or ‘for’ them” [19]. This includes any PPI including, being a co-applicant on a research project or grant application, identifying research priorities, being a member of an advisory or steering group, participating in developing research materials or giving feedback on them, conducting interviews with study participants, participating in recruitment, data collection, data analysis, drafting manuscripts and/or dissemination of results. Accordingly, we excluded studies where patients or the public were only involved as research participants.

We defined patients and public to include patients, relatives, carers, caregivers and community, which is also in line with the NIHR PPI involvement in National Health Service [19].

Patient safety included topics on medication safety, adverse events, communication, safety culture, diagnostic errors, and others. A full list of the used terms for PPI and PS is provided in Supplementary file 1. Regarding the research type and design, we included empirical qualitative, quantitative, mixed methods, and case studies. Only articles published in peer-reviewed journals and in English were included.

Any article that did not meet the inclusion criteria was excluded. Studies not reporting outcomes were excluded. Furthermore, review papers, conference abstracts, letters to editor, commentary, viewpoints, and short communications were excluded. Finally, papers published prior to 2018 were excluded.

### Study selection

The selection of eligible studies was done by the first and the second authors independently, starting with title and abstracts screening to eliminate papers that failed to meet our inclusion criteria. Then, full text screening was conducted to decide on the final included papers in this review. Covidence, an online data management system supported the review process, ensuring reviewers were blinded to each other’s decisions. Disagreements between reviewers were discussed first, in cases where the disagreement was not resolved, the fourth author was consulted.

### Data extraction and analysis

A data extraction sheet was developed using excel then piloted, discussed with the research team and modified as appropriate. The following data were extracted: citation and year of publication, objective of the study, country, PS topic, design, setting, PPI participants, PPI stages (identifying research priorities, being a member of an advisory or steering group, etc.…), frequency of PPI reporting as per the GRIPP2 checklist, and the availability of a plain language summary. Additionally, data against the five items of GRIPP2-SF (aim of PPI in the study, methods used for PPI, outcomes of PPI including the results and the extent to which PPI influenced the study overall, and reflections on PPI) were extracted. To avoid multiple publication bias and missing outcomes, data extraction was done by the first and the second authors independently and then compared. Disagreements between reviewers were first discussed, and then resolved by the third and fourth authors if needed.

### Quality assessment

The quality of PPI reporting was assessed using GRIPP2-SF developed by Staniszewska et al. [9] as it is developed to improve the quality, consistency, and reporting of PPI in social and healthcare research. Additionally the GRIPP2-SF is suitable for all studies regardless of whether PPI is the primary, secondary, or tertiary focus, whereas the GRIPP2-LF is not suitable for studies where PPI serves as a secondary or tertiary focus. The checklist includes five items (mentioned above) that authors should include in their studies. It is important to mention that Staniszewska et al. noted that “while GRIPP2-SF aims to guide consistent reporting, it is not possible to be prescriptive about the exact content of each item, as the current evidence-base is not advanced enough to make this possible” ([9] p5). For that reason, we had to develop criteria for scoring the five reporting items. We used three scoring as Yes, No, and partial for each of the five items of the GRIPP2-SF. Yes, was given when authors presented PPI information on the item clearly in the paper. No, when no information was provided, and partial when the information partially met the item requirement. For example, as per GRIPP2-SF authors should provide a clear description of the methods used for PPI in the study. In the example given by Staniszewska et al., information on patient/public partners and how many of them were provided, as well as the stages of the study they were involved in (i.e. refining the focus of the research questions, developing the search strategy, interpreting results). Thus, in our evaluation of the included studies, we gave a yes if information on PPI participants (i.e. patient partners, community partners, or family members etc..) and how many of them were involved was provided, and information on the stages or actions of their involvement in the study was provided. However, we gave a “partial” if information was not fully provided (i.e. information on patient/public partners and how many were involved in the study without describing in what stages or actions they were involved, and vice versa), and a “No” if no information was presented at all.

The quality of PPI reporting was done by the first and the second authors independently and then compared. Disagreements between reviewers were first discussed, and then resolved by the third and fourth author when needed.

Assessing the quality or risk of bias of the included studies was omitted, as the focus in this review was on appraising the quality of PPI reporting rather than assessing the quality of each research article.

### Data synthesis

After data extraction, a table summarising the included studies was developed. Studies were compared according to the main outcomes of the review; frequency of PPI reporting following the GRIPP2 checklist and the quality of reporting as per GRIPP2-SF five items, and the availability of a plain language summary.

## Results

### Search results and study selection

**Fig. 1:**
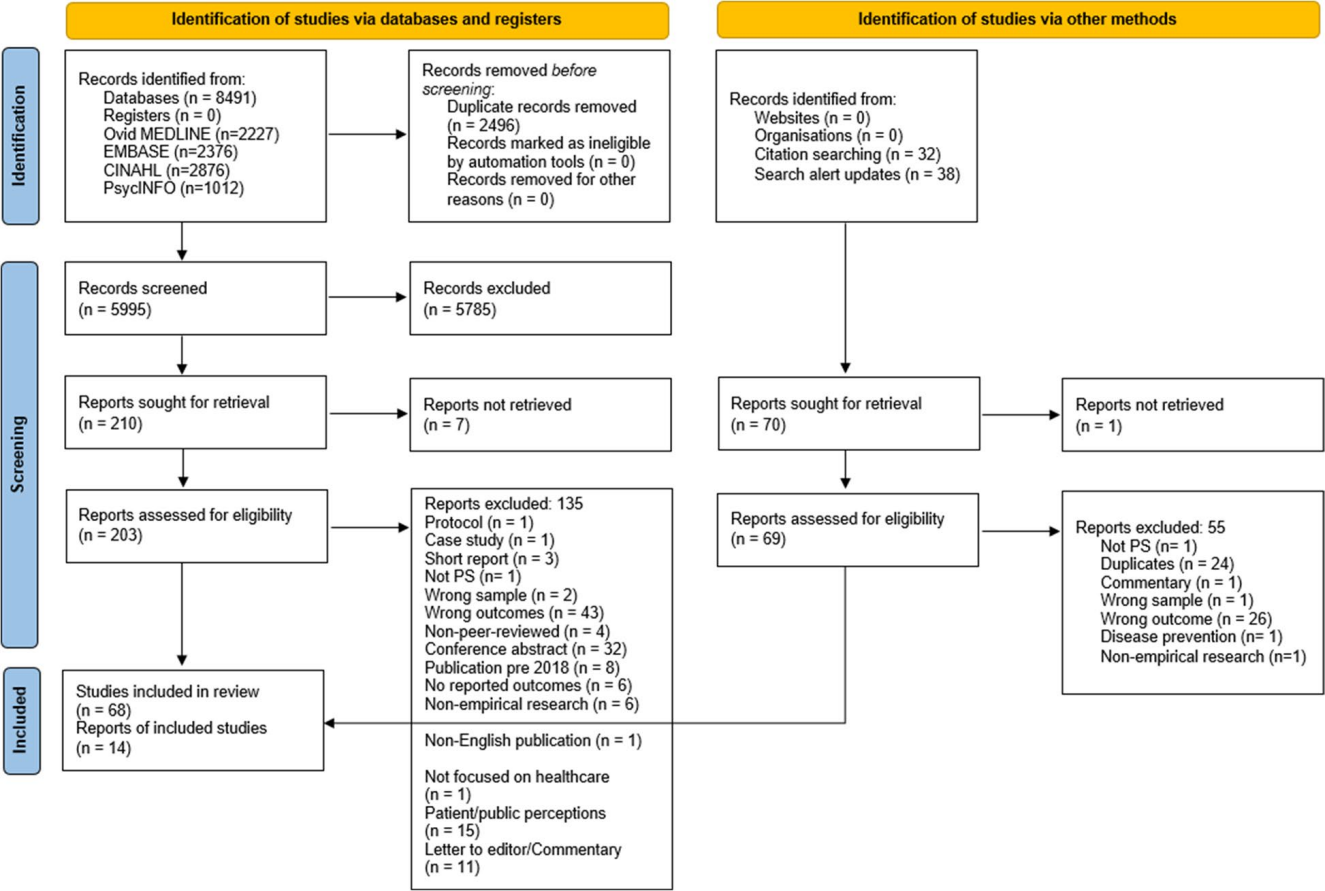
PRISMA flow diagram of the study selection process. The PRISMA flow diagram details the review search results and selection process

The database searches yielded a total of 8491 studies. First, 2496 were removed as duplicates. Then, after title and abstract screening, 5785 articles were excluded leaving 210 articles eligible for the full text review. After a careful examination, 68 of these studies were included in this review. A further 38 studies were identified from the alert system that was set on the four databases and 32 studies from the reference check of the included studies. Of these 70 articles, 56 were further excluded and 14 were added to the previous 68 included studies. Thus, 82 studies met the inclusion criteria and were included in this review. A summary of the database search results and the study selection process are presented in Fig. 1.

### Overview of included studies

Details of the study characteristics including first author and year of publication, objective, country, study design, setting, PS topic, PPI participants and involvement stages are presented in Supplementary file 2. The majority of the studies were conducted in the U.K (*n* = 24) and the United States of America (*n* = 18), with the remaining 39 conducted in other high income countries, the exception being one study in Haiti. A range of study designs were identified, the most common being qualitative (*n* = 31), mixed methods (*n* = 13), interventional (*n* = 5), and quality improvement projects (*n* = 4). Most PS topics concerned medication safety (*n* = 17), PS in general (e.g., developing a PS survey or PS management application) (*n* = 14), fall prevention (*n* = 13), communication (*n* = 11), and adverse events (*n* = 10), with the remaining PS topics listed in Supplementary file 2.

Patient representatives, advocates, and patient advisory groups (*n* = 33) and patients, service users, and health consumers (*n* = 32) were the main groups involved. The remaining, included community members/ organisations. Concerning PPI stages, the main involvement across the studies was in commenting on or developing research materials (*n* = 74) including, patient leaflets, interventional tools, mobile applications, and survey instruments. Following this stage, involvement in data analysis, drafting manuscripts, and disseminating results (*n* = 30), and being a member of a project advisory or steering group (*n* = 18) were the most common PPI evident in included studies. Whereas the least involvement was in identifying research priorities (*n* = 5), and being a co-applicant on a research project or grant application (*n* = 6).

Regarding plain language summary, only one out of the 82 studies (1.22%) provided a plain language summary in their paper [20].

### Frequency and quality of PPI reporting

The frequency of PPI reporting following the GRIPP2 checklist was 6.1%, where only five of the 82 included studies reported PPI in their papers following the GRIPP2 checklist. The quality of PPI reporting in those studies is presented in Table 2. Of these five studies, one study (20%) did not report the aim of PPI in the study and one (20%) did not comment on the extent to which PPI influenced the study overall.

**Table 2:** Quality of PPI reporting of included studies that followed GRIPP2 checklist

Publication	Report the aim of PPI in the study	Provide a clear description of the methods used for PPI in the study	Outcomes-Report the results of PPI in the study, including both positive and negative outcomes	Outcomes-Comment on the extent to which PPI influenced the study overall. Describe positive and negative effects	Comment critically on the study, reflecting on the things that went well and those that did not, so others can learn from this experience
Bisset et al. 2020 [21]	Yes	Yes	Yes	No	Yes
Morris et al. 2021 [22]	Yes	Yes	Yes	Yes	Yes
Tobiano et al. 2022 [23]	No	Yes	Yes	Yes	Yes
Francis-Coad et al. 2023 [24]	Yes	Yes	Yes	Yes	Yes
Troya et al. 2019 [25]	Yes	Yes	Yes	Yes	Yes

*PPI* patient and public involvement, *GRIPP2* Guidance for Reporting Involvement of Patients and the Public

The quality of PPI reporting of the remaining 77 studies is presented in Table 3. The aim of PPI in the study was reported in 62.3% of articles (*n* = 48), while 3.9% (*n* = 3) partially reported this. A clear description of the methods used for PPI in the study was reported in 79.2% of papers (*n* = 61) and partially in 20.8% (*n* = 16). Concerning the outcomes, 81.8% of papers (*n* = 63) reported the results of PPI in the study, while 10.4% (*n* = 8) partially did. Of the 77 studies, 68.8% (*n* = 53) reported the extent to which PPI influenced the study overall and 3.9% (*n* = 3) partially reported this. Finally, 57.1% (*n* = 44) of papers critically reflected on the things that went well and those that did not and 2.6% (*n* = 2) partially reflected on this.

**Table 3:** Quality of PPI reporting following the criteria of the GRIPP2-SF checklist within the included studies

Publication	Report the aim of PPI in the study	Provide a clear description of the methods used for PPI in the study	Outcomes-Report the results of PPI in the study, including both positive and negative outcomes	Outcomes-Comment on the extent to which PPI influenced the study overall. Describe positive and negative effects	Comment critically on the study, reflecting on the things that went well and those that did not, so others can learn from this experience
Aharaz et al. 2023 [26]	Partial	Yes	Yes	No	Yes
Aho-Glele et al. 2021 [27]	Partial	Yes	Yes	No	Partial
Albutt et al. 2020 [28]	No	Yes	Yes	Yes	No
Bell et al. 2022 [29]	Yes	Yes	Yes	Yes	Yes
Boet et al. 2021 [30]	No	Yes	Yes	Yes	No
Carter et al. 2018 [31]	No	Partial	Partial	No	No
Da Silva Lopes et al. 2021 [32]	No	Yes	Yes	Yes	Yes
de Jong et al. 2019 [33]	Yes	Yes	Yes	Yes	Yes
Doucette et al. 2023 [34]	Yes	Partial	Yes	Yes	Yes
Elrod et al. 2023 [35]	Yes	Yes	Yes	Yes	Yes
Feldman et al. 2023 [36]	No	Yes	Yes	Yes	No
Francis-Coad et al. 2022 [37]	Yes	Yes	Yes	Yes	Yes
Fuller et al. 2020 [38]	Yes	Yes	Yes	Yes	No
Gibson et al. 2020 [39]	Yes	Partial	Yes	No	Yes
Giles et al. 2020 [40]	Yes	Yes	Yes	Yes	No
Gnagi et al. 2022 [41]	No	Yes	Yes	Partial	No
Goodsmith et al. 2021 [42]	Yes	Yes	Yes	Yes	Yes
Gorman et al. 2023 [43]	Yes	Yes	Partial	Yes	No
Green et al. 2021 [44]	Yes	Yes	Yes	Yes	Yes
Guo et al. 2023 [45]	No	Yes	No	Yes	No
Hahn-Goldberg 2022 [46]	Yes	Yes	Yes	Yes	Yes
Harrington et al. 2019 [47]	Yes	Yes	Yes	Yes	No
Harris et al. 2022 [48]	Yes	Yes	Yes	Yes	No
Hawley-Hague et al. 2020 [49]	Yes	Yes	Yes	Yes	Yes
Holmqvist et al. 2023 [50]	Yes	Yes	Yes	Yes	Yes
Jayesinghe et al. 2022 [51]	No	Partial	Partial	Yes	No
Johannessen et al. 2019 [52]	No	Yes	Yes	Yes	Yes
Joseph et al. 2022 [53]	No	Partial	Partial	No	No
Khan et al. 2018 [54]	Yes	Partial	No	Yes	No
Khazen et al. 2023 [55]	No	Yes	Yes	No	Yes
Knight et al. 2019 [56]	No	Partial	No	Yes	No
Lawrence et al. 2019 [57]	No	Yes	Yes	No	Yes
Louch et al. 2019 [58]	No	Yes	Yes	Yes	Yes
MacDonald et al. 2018 [59]	Yes	Yes	Yes	Yes	Yes
Mackintosh et al. 2018 [60]	Yes	Yes	Yes	Yes	Yes
Marchand et al. 2022 [61]	Yes	Yes	Yes	Yes	Yes
Mazuz & Biswas 2022 [62]	Yes	Yes	Yes	Yes	Yes
McCahon et al. 2022 [63]	No	Yes	Yes	No	Yes
McMullen et al. 2023 [64]	Yes	Yes	Yes	Partial	Yes
Morris et al. 2023 [65]	No	Partial	No	No	No
Morris et al. 2018 [66]	Yes	Yes	Yes	No	Yes
Nether et al. 2022 [67]	No	Yes	Partial	Yes	No
Powell et al. 2021 [68]	Yes	Yes	Yes	Yes	Yes
Powell et al. 2022 [69]	No	Partial	Partial	Yes	No
Radecki et al. 2020 [70]	Yes	Partial	Partial	Yes	Yes
Rosgen et al. 2022 [71]	Partial	Yes	Yes	Yes	No
Schenk et al. 2019 [72]	Yes	Yes	Yes	Yes	Yes
Shahid et al. 2022 [20]	Yes	Yes	Yes	Yes	No
Spazzapan et al. 2020 [73]	Yes	Partial	Partial	Yes	No
Stoll et al. 2021 [74]	Yes	Yes	Yes	Yes	Yes
Subbe et al. 2021 [75]	Yes	Partial	No	No	No
Tai et al. 2020 [76]	Yes	Yes	Yes	Yes	No
Thakur et al. 2021 [77]	Yes	Yes	Yes	Partial	No
Thomas et al. 2021 [78]	Yes	Yes	Yes	Yes	Yes
Tremblay et al. 2021 [79]	Yes	Yes	Yes	No	Yes
Troya et al. 2019 [80]	No	Partial	Yes	Yes	No
Tyler et al. 2021 [81]	Yes	Yes	Yes	Yes	Yes
Tyler et al. 2023 [82]	No	Yes	Yes	Yes	Yes
Van den Bulck et al. 2020 [83]	Yes	Yes	Yes	Yes	Partial
Van Strien‐Knippenberg et al. 2022 [84]	Yes	Yes	Yes	No	Yes
Wilson et al. 2021 [85]	No	Partial	Yes	No	No
Winterberg et al. 2022 [86]	Yes	Partial	Yes	Yes	Yes
Yang et al. 2020 [87]	Yes	Yes	Yes	No	No
Young et al. 2018 [88]	Yes	Yes	Yes	No	Yes
Yuen et al. 2023 [89]	No	Yes	Yes	No	Yes
Jo & Nabatchi 2019 [90]	Yes	Yes	Yes	Yes	Yes
O'Hara et al. 2018 [91]	Yes	Yes	Yes	Yes	Yes
de Jong et al. 2019 [92]	Yes	Yes	Yes	Yes	Yes
O'Donnell et al. 2019 [93]	Yes	Yes	Yes	Yes	Yes
Russ et al. 2020 [94]	Yes	Yes	Yes	Yes	No
Mazuz et al. 2020 [95]	No	Yes	Yes	No	No
Hjelmfors et al. 2018 [96]	No	Yes	Yes	Yes	Yes
Horgan et al. 2023 [97]	No	Partial	No	No	No
Tyler et al. 2020 [98]	No	Yes	Yes	No	Yes
Ward et al. 2018 [99]	Yes	Yes	Yes	Yes	Yes
Berthelsen et al. 2023 [100]	Yes	Yes	Yes	No	No
Okkenhaug et al. 2023 [101]	Yes	Yes	Yes	Yes	Yes

*PPI* patient and public involvement, *GRIPP2* Guidance for Reporting Involvement of Patients and the Public

## Discussion

### Summary of main findings

This systematic review assessed the frequency of reporting PPI in PS research using the GRIPP2 checklist and quality of reporting using the GRIPP2-SF. In total, 82 studies were included in this review. Major PS topics were related to medication safety, general PS, and fall prevention. Patient representatives, advocates, patient advisory groups, patients, service users, and health consumers were the most involved. The main involvement across the studies was in commenting on or developing research materials such as educational and interventional tools, survey instruments, and applications while the least was in identifying research priorities and being a co-applicant on a research project or grant application. Thus, significant effort is still needed to involve patients and the public in the earlier stages of the research process given the fundamental impact of PS on their lives.

### Overall completeness and applicability of evidence

A low frequency of reporting PPI in PS research following the GRIPP2 guidelines was revealed in this review, where only five of the 82 studies included mentioned that PPI was reported as per the GRIPP2 checklist. This is despite it being the most recent report-focused framework and the most recommended by several leading journals [17]. This was not surprising as similar results were reported in recent reviews in other healthcare topics. For instance, Musbahi et al. in their systematic review on PPI reporting in bariatric research reported that none of the 90 papers identified in their review mentioned or utilised the GRIPP2 checklist [102]. Similarly, a study on PPI in orthodontic research found that none of the 363 included articles reported PPI against the GRIPP2 checklist [103].

In relation to the quality of reporting following the GRIPP2-SF criteria, our findings show sub-optimal reporting within the 77 studies that did not use GRIPP2 as a guide/checklist to report their PPI. Similarly, Bergin et al. in their systematic review to investigate the nature and impact of PPI in cancer research concluded that substandard reporting was evident [12]. In our review, this was mainly due to failure to meet three criteria. First, the lowest percentage of reporting (57.1%, *n* = 44) was related to critical reflection on PPI in the study (i.e., what went well and what did not). In total, 31 studies (42.9%) did not provide any information on this, and two studies were scored as partial. The first study mentioned that only involving one patient was a limitation [27] and the other stated that including three patients in the design of the tool was a strength [83]. Both studies did not critically comment or reflect on these points so that future researchers are able to avoid such problems and enhance PPI opportunities. For instance, providing the reasons/challenges behind the exclusive inclusion of a single patient and explaining how this limits the study findings and conclusion would help future researchers to address these challenges. Likewise, commenting on why incorporating three patients in the design of the study tool could be seen as a strength would have been beneficial. This could be, fostering diverse perspectives and generating novel ideas for developing the tool. Similar to our findings, Bergin et al. in their systematic review reported that 40% of the studies failed to meet this criterion [12].

Second, only 48 out of 77 articles (62.3%) reported the aim of PPI in their study, which is unlike the results of Bergin et al. where most of the studies (93.1%) in their review met this criterion [12]. Of the 29 studies which did not meet this criterion in our review, few mentioned in their objective developing a consensus-based instrument [41], reaching a consensus on the patient-reported outcomes [32], obtaining international consensus on a set of core outcome measures [98], and facilitating a multi-stakeholder dialogue [71] yet, without indicating anything in relation to patients, patient representatives, community members, or any other PPI participants. Thus, the lack of reporting the aim of PPI was clearly evident in this review. Reporting the aim of PPI in the study is crucial for promoting transparency, methodological rigor, reproducibility, and impact assessment of the PPI.

Third, 68.8% (*n* = 53) of the studies reported the extent to which PPI influenced the study overall including positive and negative effects if any. This was again similar to the findings of Bergin et al., where 38% of the studies did not meet this criterion mainly due to a failure to address PPI challenges in their respective studies [12]. Additionally, Owyang et al. in their review on the extent, and quality of PPI in orthopaedic practice, also described a poor reporting of PPI impact on research [13]. As per the GRIPP2 guidelines, both positive and negative effects of PPI on the study should be reported when applicable. Providing such information is essential as it enhances future research on PPI in terms of both practice and reporting.

Reporting a clear description of the methods used for PPI in the study was acceptable, with 79.2% of the papers meeting this criterion. Most studies provided information in the methods section of their papers on the PPI participants, their number, stages of their involvement and how they were involved. Providing clear information on the methods used for PPI is vital to give the reader a clear understanding of the steps taken to involve patients, and for other researchers to replicate these methods in future research. Additionally, reporting the results of PPI in the study was also acceptable with 81.8% of the papers reporting the outcomes of PPI in the results section. Reporting the results of PPI is important for enhancing methodological transparency, providing a more accurate interpretation for the study findings, contributing to the overall accountability and credibility of the research, and informing decision making.

Out of the 82 studies included in this review, only one study provided a plain language summary. We understand that PS research or health and medical research in general is difficult for patients and the public to understand given their diverse health literacy and educational backgrounds. However, if we expect patients and the public to be involved in research then, it is crucial to translate this research that has a huge impact on their lives into an easily accessible format. Failing to translate the benefits that such research may have on patient and public lives may result in them underestimating the value of this research and losing interest in being involved in the planning or implementation of future research [103]. Thus, providing a plain language summary for research is one way to tackle this problem. To our knowledge, only a few health and social care journals (i.e. Cochrane and BMC Research Involvement and Engagement) necessitate a plain language summary as a submission requirement. Having this as a requirement for submission is crucial in bringing the importance of this issue to researchers’ attention.

Research from recent years suggests that poor PPI reporting in articles relates to a lack of submission requirements for PPI reporting in journals and difficulties with word limits for submitted manuscripts [13]. Price et al. assessed the frequency of PPI reporting in published papers before and after the introduction of PPI reporting obligations by the British Medical Journal (BMJ) [104]. The authors identified an increase in PPI reporting in papers published by BMJ from 0.5% to 11% between the periods of 2013–2014 and 2015–2016. The study findings demonstrate the impact of journal guidelines in shaping higher quality research outputs [13]. In our review, we found a low frequency of PPI reporting in PS research using the GRIPP2 checklist, alongside sub-optimal quality of reporting following GRIPP2-SF. This could potentially be attributed to the absence of submission requirements for PPI reporting in journals following the GRIPP2 checklist, as well as challenges posed by word limits.

### Strengths and limitations

This systematic review presents an overview on the frequency of PPI reporting in PS research using the GRIPP2 checklist, as well as an evaluation of the quality of reporting following the GRIPP2-SF. As the first review to focus on PS research, it provides useful knowledge on the status of PPI reporting in this field, and the extent to which researchers are adopting and adhering to PPI reporting guidelines. Despite these strengths, our review has some limitations that should be mentioned. First, only English language papers were included in this review due to being the main language of the researchers. Thus, there is a possibility that relevant articles on PPI in PS research may have been omitted. Another limitation is related to our search which was limited to papers published starting 2018 as the GRIPP2 guidelines were published in 2017. Thus it is probable that the protocols of some of these studies were developed earlier than the publication of the GRIPP2 checklist, meaning that PPI reporting following GRIPP2 was not common practice and thus not adopted by these studies. This might limit the conclusions we can draw from this review. Finally, the use of GRIPP2 to assess the quality of PPI reporting might be a limitation as usability testing has not yet been conducted to understand how the checklist works in practice with various types of research designs. However, the GRIPP2 is the first international, evidence-based, community consensus informed guideline for the reporting of PPI in health and social care research. Reflections and comments from researchers using the GRIPP2 will help improve its use in future studies.

### Implications for research and practice

Lack of PPI reporting not only affects the quality of research but also implies that others cannot learn from previous research experience. Additionally, without consistent and transparent reporting it is difficult to evaluate the impact of various PPI in research [9]: “if it is not reported it cannot be assessed” ([105] p19). Enhanced PPI reporting will result in a wider range and richer high-quality evidence-based PPI research, leading to a better understanding of PPI use and effectiveness [103]. GRIPP2 reporting guidelines were developed to provide guidance for researchers, patients, and the public to enhance the quality of PPI reporting and improve the quality of the international PPI evidence-base. The guidance can be used prospectively to plan PPI or retrospectively to guide the structure or PPI reporting in research [9]. To enhance PPI reporting, we recommend the following;

#### Publishers and journals

First, we encourage publishers and journals to require researchers to report PPI following the GRIPP2 checklist. Utilising the short or the long version should depend on the primary focus of the study (i.e., if PPI is within the primary focus of the research then the GRIPP2-LF is recommended). Second, we recommend that journals and editorial members advise reviewers to evaluate PPI reporting within research articles following the GRIPP2 tool and make suggestions accordingly. Finally, we encourage journals to add a plain language summary as a submission requirement to increase research dissemination and improve the accessibility of research for patients and the public.

#### Researchers

Though there is greater evidence of PPI in research, it is still primarily the researchers that are setting the research agenda and deciding on the research questions to be addressed. Thus, significant effort is still needed to involve patients and the public in the earlier stages of the research process given the fundamental impact of PS on their lives. To enhance future PPI reporting, perhaps adding a criterion following the GRIPP2 tool to existing EQUATOR checklists for reporting research papers such as STROBE, PRISMA, CONSORT, may support higher quality research. Additionally, currently, there is no detailed explanation paper for the GRIPP2 where each criterion is explained in detail with examples. Addressing this gap would be of great benefit to guide the structure of PPI reporting and to explore the applicability of each criterion in relation to different stages of PPI in research. For instance, having a detailed explanation for each criterion across different research studies having various PPI stages would be of high value to improve future PPI reporting given the growing interest in PPI research in recent years and the relatively small PPI evidence base in health and medical research.

#### Funders

Funding bodies can also enhance PPI reporting by adding a requirement for researchers to report PPI following the GRIPP2 checklist. In Ireland, the National HSE has already initiated this by requiring all PPI in HSE research in Ireland to be reported following the GRIPP2 guidelines [10].

## Conclusions

This study represents the first systematic review on the frequency and quality of PPI reporting in PS research using the GRIPP2 checklist. Most PS topics were related to medication safety, general PS, and fall prevention. The main involvement across the studies was in commenting on or developing research materials. Thus, efforts are still needed to involve patients and the public across all aspects of the research process, especially earlier stages of the research cycle. The frequency of PPI reporting following the GRIPP2 guidelines was low, and the quality of reporting following the GRIPP2-SF criteria was sub-optimal. The lowest percentages of reporting were on critically reflecting on PPI in the study so future research can learn from this experience and work to improve it, reporting the aim of the PPI in the study, and reporting the extent to which PPI influenced the study overall including positive and negative effects. Researchers, funders, publishers, journals, editorial members and reviewers have a responsibility to promote consistent and transparent PPI reporting following internationally developed reporting guidelines such as the GRIPP2. Evidence-based guidelines for reporting PPI should be supported to help future researchers plan and report PPI more effectively, which may ultimately improve the quality and relevance of research.

### Supplementary Information

**Supplementary Material 1. **

**Supplementary Material 2. **

## Data Availability

All data generated or analysed during this study are included in this published article and its Supplementary information files.

## Abbreviations

PS: Patient safety
U.K: United Kingdom
NIHR: National Institute for Health Research
PPI: Public and Patient Involvement
HSE: Health Service Executive
GRIPP: Guidance for Reporting Involvement of Patients and the Public
GRIPP2: Second version of the GRIPP checklist
GRIPP2-LF: Long form of GRIPP2
GRIPP2-SF: Short form of GRIPP2
PRISMA: Preferred Reporting Items for Systematic Reviews and Meta-Analyses
PROSPERO: The International Database of Prospectively Registered Systematic Reviews
BMJ: British Medical Journal
