# Supplementary material for: Systematic review on the frequency and quality of reporting patient and public involvement in patient safety research
Source: BMC Health Serv Res. 2024 Apr 26;24:532. doi: 10.1186/s12913-024-11021-z (PMC11046929; doi:10.1186/s12913-024-11021-z)
Supplement: Supplementary file 1 — Supplementary Material 1. [file 12913_2024_11021_MOESM1_ESM.docx]

**Supplementary file 1**

**Ovid MEDLINE Search Strategy**

| **#** | **Searches** | **Results** |
| --- | --- | --- |
| 1 | Patient Participation/ | 29409 |
| 2 | Community Participation/ | 18448 |
| 3 | ((patient$ or public or communit* or citizen$ or famil* or carer$ or caregiver$ or relative$ or consumer$ or user$ or client$ or customer$) adj1 (empower* or involve* or engage* or participat* or consult* or partner* or collaborat* or contribut* or activat* or codesign* or co-design* or coproduc* or co-produc*)).tw. | 122964 |
| 4 | 1 or 2 or 3 | 159282 |
| 5 | Patient Safety/ | 25412 |
| 6 | Safety Management/ | 21442 |
| 7 | exp Medical Errors/ | 121771 |
| 8 | Accident Prevention/ | 9269 |
| 9 | Patient Harm/ | 219 |
| 10 | Risk Management/ | 19466 |
| 11 | Accidental Falls/ | 28029 |
| 12 | ("patient safety" or "medica* error*" or "surgical error*" or "communication error*" or "adverse event*" or "medical harm" or "medical injur*" or "missed care" or "near miss*" or fall* or slip* or trip*).tw. | 804098 |
| 13 | 5 or 6 or 7 or 8 or 9 or 10 or 11 or 12 | 964342 |
| 14 | 4 and 13 | 6421 |
| 15 | limit 14 to yr="2018 -Current" | 2227 |

**Embase Search Strategy**

| **#** | **Searches** | **Results** |
| --- | --- | --- |
| #16 | #15 AND (2018:py OR 2019:py OR 2020:py OR 2021:py OR 2022:py OR 2023:py) | 2372 |
| #15 | #6 AND #14 | 5406 |
| #14 | #7 OR #8 OR #9 OR #10 OR #11 OR #12 OR #13 | 1244599 |
| #13 | 'patient safety':ti,ab OR 'medica* error*':ti,ab OR 'surgical error*':ti,ab OR  'communication error*':ti,ab OR 'adverse event*':ti,ab OR 'medical harm':ti,ab  OR 'medical injur*':ti,ab OR 'missed care':ti,ab OR 'near miss*':ti,ab OR fall*:ti,ab OR slip*:ti,ab OR trip*:ti,ab | 1159324 |
| #12 | 'near miss (health care)'/mj | 167 |
| #11 | 'adverse event'/mj | 26860 |
| #10 | 'accident prevention'/mj | 8694 |
| #9 | 'medical error'/mj | 8467 |
| #8 | 'safety'/mj | 58120 |
| #7 | 'patient safety'/mj | 27109 |
| #6 | #1 OR #2 OR #3 OR #4 OR #5 | 115678 |
| #5 | ((patient? OR public OR communit* OR citizen? OR famil* OR carer? OR caregiver? OR relative? OR consumer? OR user? OR client? OR customer?) NEAR/1 (empower* OR involve* OR engage* OR participat* OR consult* OR partner* OR collaborat* OR contribut* OR activat* OR codesign* OR 'co design*' OR coproduc* OR 'co produc*')):ti,ab | 104998 |
| #4 | 'public participation'/mj | 443 |
| #3 | 'public engagement'/mj | 14 |
| #2 | 'patient engagement'/mj | 689 |
| #1 | 'patient participation'/mj | 11252 |

**CINAHL Search Strategy**

| **#** | **Searches** | **Limiters** | **Results** |
| --- | --- | --- | --- |
| S12 | S4 AND S10 | Published  Date: 20180101-  20231231 | 2,872 |
| S11 | S4 AND S10 |  | 6,149 |
| S10 | S5 OR S6 OR S7 OR S8 OR S9 |  | 342,812 |
| S9 | TI ( ('patient safety' OR 'medica* error*' OR 'surgical error*' OR 'communication error*' OR 'adverse event*' OR 'medical harm' OR 'medical injur*' OR 'missed care' OR 'near miss*' OR fall* OR slip* OR trip*) ) OR AB ( ('patient safety' OR 'medica* error*' OR 'surgical error*' OR 'communication error*' OR 'adverse event*' OR 'medical harm' OR 'medical injur*' OR 'missed care' OR 'near miss*' OR fall* OR slip* OR trip*) ) |  | 236,573 |
| S8 | (MH "Risk Control and Safety (Iowa NOC)+") |  | 16 |
| S7 | (MH "Risk Management (Iowa NIC)+") |  | 32 |
| S6 | (MM "Accidental Falls") |  | 16,158 |
| S5 | (MH "Patient Safety+") |  | 145,405 |
| S4 | S1 OR S2 OR S3 |  | 102,888 |
| S3 | AB ( (Patient? OR public OR communit* OR citizen? OR famil* OR carer? OR caregiver? OR relative? OR consumer? OR user? OR client? OR customer?) N1 (Empower* OR Involve* OR Engage* OR participat* OR consult* OR partner* OR collaborat* OR contribut* OR activat* OR codesign* OR co-design* OR coproduc* OR co-produc*) ) |  | 84,702 |
| S2 | TI ( (Patient? OR public OR communit* OR citizen? OR famil* OR carer? OR caregiver? OR relative? OR consumer? OR user? OR client? OR customer?) N1 (Empower* OR Involve* OR Engage* OR participat* OR consult* OR partner* OR collaborat* OR contribut* OR activat* OR codesign* OR co-design* OR coproduc* OR co-produc*) ) |  | 17,753 |
| S1 | (MM "Consumer Participation") |  | 13,962 |

**PsycINFO Search Strategy**

| **#** | **Searches** | **Limiters** | **Results** |
| --- | --- | --- | --- |
| S9 | S4 AND S7 | Publication Year: 2018-2023 | 1,012 |
| S8 | S4 AND S7 |  | 2,639 |
| S7 | S5 OR S6 |  | 113,015 |
| S6 | TI ( ('patient safety' OR 'medica* error*' OR 'surgical error*' OR 'communication error*' OR 'adverse event*' OR 'medical harm' OR 'medical injur*' OR 'missed care' OR 'near miss*' OR fall* OR slip* OR trip*) ) OR AB ( ('patient safety' OR 'medica* error*' OR 'surgical error*' OR 'communication error*' OR 'adverse event*' OR 'medical harm' OR 'medical injur*' OR 'missed care' OR 'near miss*' OR fall* OR slip* OR trip*) ) |  | 105,094 |
| S5 | DE "Patient Safety" OR DE "Falls" OR DE "Risk Management" |  | 14,820 |
| S4 | S1 OR S2 OR S3 |  | 92,357 |
| S3 | AB ((Patient? OR public OR communit* OR citizen? OR famil* OR carer? OR caregiver? OR relative? OR consumer? OR user? OR client? OR customer?) N1 (Empower* OR Involve* OR Engage* OR participat* OR consult* OR partner* OR collaborat* OR contribut* OR activat* OR codesign* OR co-design* OR coproduc* OR co-produc*)) |  | 86,103 |
| S2 | TI ((Patient? OR public OR communit* OR citizen? OR famil* OR carer? OR caregiver? OR relative? OR consumer? OR user? OR client? OR customer?) N1 (Empower* OR Involve* OR Engage* OR participat* OR consult* OR partner* OR collaborat* OR contribut* OR activat* OR codesign* OR co-design* OR coproduc* OR co-produc*)) |  | 13,096 |
| S1 | DE "Client Participation" OR DE "Patient Centered Care" |  | 3,839 |
