# Supplementary material for: Systematic review on the frequency and quality of reporting patient and public involvement in patient safety research
Source: BMC Health Serv Res. 2024 Apr 26;24:532. doi: 10.1186/s12913-024-11021-z (PMC11046929; doi:10.1186/s12913-024-11021-z)
Supplement: Supplementary file 2 — Supplementary Material 2. [file 12913_2024_11021_MOESM2_ESM.docx]

**Supplementary file 2**

Basic characteristics of included studies.

| **Publication** | **Objective** | **Country** | **Study type/ design** | **Setting** | **Patient safety topic** | **PPI participants** | **Involvement stage** |
| --- | --- | --- | --- | --- | --- | --- | --- |
| Aharaz et al. 2023 | Utilise participatory research to examine the issues with the current shared medical record within a Danish hospital and develop a prototype that addresses these issues | Denmark | Qualitative and participatory design | Hospital | Medication safety | Patients | 4 |
| Aho-Glele et al. 2021 | Present the development of a pan-Canadian survey tool to be used by subject matter experts in patient engagement and patient safety | Canada | Quantitative | Healthcare  organisations | Patient safety | Patient representative | 3 |
| Albutt et al. 2020 | Develop and evaluate the feasibility of an approach to involving patients and relatives in the detection of clinical deterioration in hospital | England | Mixed methods | Hospital | Communication | Patient representatives | 4 |
| Bell et al. 2022 | Develop a framework with patients/ families to help organisations identify and categorise patient-reported diagnostic process-related breakdowns (PRDBs) to inform organisational learning | USA | Qualitative | Hospital | Diagnostic errors | Patients and family members | 3,4 |
| Bisset et al. 2020 | To explore whether the personality of the surgeon was important to patients, and whether the surgeon’s personality influenced shared decision-making | U.K | Mixed methods | Single centre colorectal database | Communication | Patient representatives | 4,5,6 |
| Boet et al. 2021 | Contribute to the development of evidence-based best practices for implementing innovative tools to directly assess performance in the operating room, using the case of the Operating Room Black Box | Canada | Qualitative | Hospital | Adverse events | Patients and patient advisors | 1,4 |
| Carter et al. 2018 | Explore the experience and views of women with threatened preterm labour symptoms and the factors that could both positively and negatively affect that experience | U.K | Qualitative | Hospital | Preterm labour | Patient and public panel | 3,6 |
| Da Silva Lopes et al. 2021 | Reach a consensus on the patient-reported outcomes (PROs) version of the Common Terminology Criteria for Adverse Events Symptom Terms relevant for cancer patients treated with immune checkpoint inhibitors (ICIs) and gather preliminary expert opinions on additional PRO symptom terms that could be related to symptomatic ICI toxicity | European countries | Delphi method | Hospital | Adverse events | Patient advocate | 3,4 |
| de Jong et al. 2019 | Design a series of audio‐visual (AV) falls prevention messages in collaboration with community‐dwelling older people and explore the messages’ effect on older people's knowledge, awareness, and motivation to take action regarding falls prevention | Australia | Exploratory community‐based participatory study | Community | Fall prevention | Older people | 3,4 |
| Doucette et al. 2023 | Describe the findings from a community-engaged approach to building novel solutions to improve medication reconciliation among racially diverse older adults | USA | Participatory design | Community | Medication safety | Community-engaged  research group and Community-dwelling adults | 2,3,4 |
| Elrod et al. 2023 | Describes the development of  a collaboration between a university and local community-based organisations to adopt and implement multiple evidence-based fall prevention programs, each one targeting older adults at different levels of fall risk, in a large metropolitan setting | USA | Case study | Community | Fall prevention | Community organisations and volunteer community advocates | 1,3 |
| Feldman et al. 2023 | Develop a standardised Patient Reported Outcomes Common Terminology Criteria for Adverse Events item subset for unambiguously monitoring adverse events in prostate cancer patient | The Netherlands | Mixed methods | Hospital and community | Adverse events | Patients | 6 |
| Francis-Coad et al. 2022 | Co-design a falls prevention education programme with aged care home residents and staff and evaluate its feasibility | Australia and Wales | Mixed methods | Aged care homes | Fall prevention | Aged care home residents | 3,4 |
| Fuller et al. 2020 | Evaluate the implementation of a suite of digital health tools integrated with the electronic health record to engage hospitalised patients, caregivers, and their care team in preparing for discharge | USA | Mixed methods | Medical centre | Discharge communication | Patients and Patient and family advisory council | 4 |
| Gibson et al. 2020 | Engage patients and informal caregivers in co-designing an electronic application that would integrate the functionality of the Patient Safety Active Management system with the information provided by the patient safety guides into a dashboard accessed through a patient portal | USA | User centered design sessions | Hospital | Patient safety | Patients and caregivers | 4 |
| Giles et al. 2020 | Develop a patient-centered contributory factors framework and implementation checklist for examining medication safety issues that can support incident investigation and lead to improvements in understanding medication safety in primary care from the patient and carer perspective | U.K | Qualitative | Primary care | Medication safety | Patient and public involvement group | 4,5,6 |
| Gnagi et al. 2022 | Develop a consensus-based instrument to assess medication literacy of patients in the home care setting | Switzerland | Qualitative | Home health care | Medication safety | Patients | 4 |
| Goodsmith et al. 2021 | Apply a community-partnered approach to develop and implement a suicide-risk management protocol in a depression study | USA | Case study | Community | Suicide prevention | Community partners | 2,4 |
| Gorman et al. 2023 | Investigate how families can be effectively involved in supporting a patient accessing services provided by crisis resolution and home treatment teams in England, using ethnographic methods | England | Ethnographic investigation | Hospital | Suicide prevention | Patient and carer advisory panel | 3,4,5,6 |
| Green et al. 2021 | Co-design a program to help pre-frail and frail older people return to their homes following hospital discharge by increasing resilience and promoting independence | Australia | Qualitative | Hospital | Fall prevention | Patients and family members | 4 |
| Guo et al. 2023 | Investigate the effectiveness of patient engagement in fall prevention strategy in preventing falls, enhancing their level of knowledge, attitudes, and practice and fall efficacy in older patients | China | Longitudinal Quasi-experimental | Hospital | Fall prevention | Older patients | 4 |
| Hahn-Goldberg 2022 | Use participatory action research and design thinking methods to engage extreme users to understand their current experiences and design tools  to improve medication information transfer during transitions of care | Canada | Mixed methods | Hospitals and community | Medication safety | Patients and family members | 3,4,6 |
| Harrington et al. 2019 | Develop a model based on clinical engagement principles using a participatory action research framework involving staff and consumer focus groups, then evaluate its effect on rates of violence, self-harm, absconding, sexually inappropriate behaviour, and seclusion | Australia | Pre and post interventional | Psychiatric inpatient unit | Adverse events | Consumers | 4 |
| Harris et al. 2022 | Involve surgical patients in the development and validation of a safety checklist for patients to use before and after surgery | Norway | Prospective | Hospital | Surgical safety | Patients and patient representatives | 4,6 |
| Hawley-Hague et al. 2020 | Develop smartphone apps designed to support patients to exercise, based on psychological theory, and co-designed with health care professionals, older adults, and patients. | U.K | Qualitative | Community | Fall prevention | Patients and older adults | 4 |
| Holmqvist et al. 2023 | Describe how remote co-design was applied to create a medication plan prototype and to explore participants’ experiences with this approach | Sweden | Case study | Home health care | Medication safety | Older persons and next-of-kin | 4 |
| Jayesinghe et al. 2022 | Calculate the medication costs of potentially inappropriate prescribing for middle-aged adults according to the 22 Prescribing Optimally in Middle-aged People's Treatments criteria and compare with the cost of consensus-validated adequate alternative prescribing scenarios | U.K | Mixed methods | Community and general practice  prescribing data | Medication safety | Patient and public involvement group | 4 |
| Johannessen et al. 2019 | Develop, implement, and evaluate a research-based leadership guide for the nursing home and home care context in Norway | Norway | Qualitative | Nursing homes and home care services | Patient safety | Patient and next-of-kin representatives | 1,4 |
| Joseph et al. 2022 | Explore the suitability, feasibility and adaptability of current patient engagement strategies to enhance safety for ethnic minority consumers in cancer settings | Australia | Qualitative | Cancer settings | Adverse events | Consumer advisory group | 1 |
| Khan et al. 2018 | Determine whether patient safety, family experience, and communication processes would improve after the implementation of a programme to standardise communication with families on rounds. Families, nurses, and physicians coproduced the intervention | USA and Canada | Pre and post interventional | Paediatric inpatient units in hospitals | Communication | Parents | 4,6 |
| Khazen et al. 2023 | Identify key elements facilitating understanding and managing diagnostic uncertainty, examine optimal ways to convey uncertainty to patients, and develop and test a novel  tool to communicate diagnostic uncertainty in actual clinical encounters | USA | Qualitative | Primary care | Diagnostic errors | Patients | 4 |
| Knight et al. 2019 | To improve communication and coordination of care between settings, and engage the patient/family in the handoff, while determining if it was feasible for the team to conduct the videoconference handoff | USA | Interventional | Paediatrics units in hospitals | Bedside handover | Patient and family advisors | 4 |
| Lawrence et al. 2019 | Optimise the acceptability and feasibility of Acceptance and Commitment Therapy for older adults with treatment-resistant Generalised anxiety disorder | U.K | Qualitative | Community | Medication safety | Older people | 4 |
| Louch et al. 2019 | Produce a revised version of Patient Measure of Safety that was shorter with improved acceptability, while at the same time preserving the psychometric properties, conceptual underpinnings and diagnostic function of the longer version | U.K | Quantitative | Hospitals | Patient safety | Patient panel representatives | 4 |
| MacDonald et al. 2018 | Explore maternal mortality in rural Haiti through Community-Based Action Research. To focus on the determinants of maternal mortality from the perspectives of women of near-miss maternal experiences and community members, and their solutions to reduce maternal mortality | Haiti | Qualitative | Community | Near miss | Community members | 5,6 |
| Mackintosh et al. 2018 | Evaluate our use of the arts both as product (ie production and dissemination of the animation) and process (ie public and professional engagement in the project and their assessment of whether the product was rendered convincing by the reality of practice) | U.K | Qualitative | Community | Psychological safety | Women | 4,6 |
| Marchand et al. 2022 | Describe caregivers’ experiences accessing opioid treatment services with their young people across three communities in British Columbia. And identify opportunities and strategies for improving the quality of opioid use treatments and services for young people and respond to critical gaps in young people’s access to developmentally appropriate and family-centred opioid treatment services | Canada | Qualitative | Community | Medication safety | Caregivers and community-based family peer-support team | 4,6 |
| Mazuz & Biswas 2022 | Build upon conceptual and theoretical findings regarding co-design and the aging and technology model to offer an interpretive framework to contribute to our understanding of the dynamic and innovative relations between aging and technology that arise during co-design interactions | Israel | Qualitative | Community | Fall prevention | Older adults | 4 |
| McCahon et al. 2022 | Gain a better understanding of patient perceptions and experiences of medication review as undertaken in routine general practice, including the processes and activities that led up to and shaped the review | U.K | Qualitative | Community | Medication safety | Patient and public involvement advisers | 4 |
| McMullen et al. 2023 | Conducted a workshop that brought together patients and carers as well as expertise academics to identify problems and solutions to inform future carer‐led patient safety interventions after hospital discharge as well as interventions to support the carer's well‐being during this challenging time | U.K | Nominal group technique | Mental health services and community | Patient safety | Patients and carers | 4 |
| Morris et al. 2023 | Explore patients' and carers' views of being involved in patient safety in primary care and their views of potentially using the patient safety guide for primary care to support involvement | U.K | Qualitative | Primary care | Patient safety | Patients, carers and patient and public involvement group | 4,6 |
| Morris et al. 2021 | To co-design a patient safety guide for primary care to support patients and carers to address key patient safety questions and identify key points where they can make their care safer | U.K | Participatory design | Primary care | Patient safety | Patients, carers and patient and public involvement group | 4,5,6 |
| Morris et al. 2018 | Identify unanswered research questions in the field of primary care patient safety research and to identify the top research priorities for primary care patient safety from patients, carers and primary care healthcare professionals | U.K | James Lind Alliance Priority Setting Partnership  approach | Primary care | Patient safety | Patient representatives | 2,3,4,6 |
| Nether et al. 2022 | Describes how a Neonatal Intensive Care Unit (NICU) implemented an Robust Process Improvement program including the provision of leadership support, the methods for training and mentoring of staff and parents, and the sustained outcomes to date for the improvement initiatives completed as part of the training | USA | Robust Process Improvement project | NICU of Children hospital | Central line-associated blood  stream infections | Parent advisory council members | 3,4 |
| Powell et al. 2021 | Develop a method of meaningfully involving patients as analysts of patient interviews in a process evaluation | U.K | Qualitative | Hospital and community | Medication safety | Patient-led steering group | 3,4,6 |
| Powell et al. 2022 | Understand the implementation and experience of the Medicines at Transitions of care Intervention (MaTI) from the patient perspective. And present the findings from the process evaluation patient interviews and triangulate with the trial data on MaTI adherence | U.K | Qualitative | Hospital | Medication safety | Patient-led steering group | 3,6 |
| Radecki et al. 2020 | Evaluate the impact of the patient fall self-assessment tool (specific to the inpatient setting engaging patients to coproduce the fall prevention plan) on patient knowledge in action, its usability as rated by nurses, and incidence of falls | USA | Quality improvement project | Trauma centre | Fall prevention | Patients | 4 |
| Rosgen et al. 2022 | Evaluate existing patient- and family-centered transitions in care tools, facilitate multi-stakeholder dialogue on the overall assessment and modifications required to make the tools applicable to a critically ill adult population, and identify facilitators, barriers, and implementation considerations for a transition in care bundle | Canada | Mixed methods | Hospital | Adverse events | Patient partners and family members | 4 |
| Schenk et al. 2019 | Describe the process used to engage patients and family members in the Patient-Centered Outcomes Research Institute-like patient-centered outcomes research focusing on reducing patient harm; and describe findings from a qualitative study exploring the attitudes and perceptions of patients, family members, and multidisciplinary clinical staff toward the feasibility of an intervention proposed by the pre-research team, as an approach to increasing patient/family engagement in reducing patient harm | USA | Qualitative | Hospital | Adverse events | Patients and family members | 3,4,5,6 |
| Shahid et al. 2022 | Adapt the content of the patient-oriented discharge summary tool (PODS) to the intensive care unit (ICU) context based on input from key stakeholder groups including patient partners, clinicians, and researchers; pilot test the adapted PODS-ICU in the ICU to determine its acceptability and feasibility; and gather patient, family-caregiver, and clinician perspectives on the usability of the tool and quality of information provided to patients and family-caregivers during a discharge from the ICU | Canada | Quality improvement project | ICUs of two hospitals | Discharge communication | Patient partners (patient and caregiver) | 4,6 |
| Spazzapan et al. 2020 | Investigate the benefits of introducing personalised bedside boards that focus on nonmedical information in Paediatric intensive care units (PICU). We hypothesise that the introduction of such boards would help improve staff’s knowledge of patients as individuals, as well as increase patient and family involvement in the provision of humanised care, in turn improving patient-centered care | U.K | Quality improvement project | PICU of a hospital | Patient safety | Parents | 4 |
| Stoll et al. 2021 | Describe the development of three novel patient-driven deprescribing animated videos designed to educate older adults and their caregivers about unsafe prescribing and medication harms, and to empower them to initiate deprescribing conversations with their healthcare providers | USA | Community based participatory research | Community | Medication safety | Older patients, caregivers, and Elder Voices group | 4,6 |
| Subbe et al. 2021 | Develop and test novel documentation formats to support patient contribution to their own health records during emergency hospital admissions | U.K | Mixed methods | Medical unit in a hospital | Patient safety | Patient representatives | 4 |
| Tai et al. 2020 | Explore Falls Prevention Clinic participants’ perspectives on patient-reported outcome measures and on how these measures might impact their own adherence to recommendations using a patient oriented qualitative study design with patient partners in British Columbia, Canada | Canada | Qualitative | Primary care | Fall prevention | Patient partners | 1,4,5,6 |
| Thakur et al. 2021 | Explore Paediatric caregivers’ baseline knowledge as well as their expectations for opioid medication consults in order to refine an opioid safety consultation intervention; and  explore their post-consult knowledge and evaluation after they received the refined opioid safety consultation | USA | Pre and post interventional | Outpatient pharmacy at a children’s hospital | Medication safety | Caregivers | 4 |
| Thomas et al. 2021 | Identify key health consumer perspectives and experiences based on the challenges they have faced with test-results management; engage health consumers in qualitative health services research; and provide a forum where health consumers can participate in qualitative research to generate key themes related to test-results management based on consumer selected priority topic areas | Australia | Qualitative | Emergency departments of public hospitals | Missed care | Health consumer volunteers | 4,6 |
| Tobiano et al. 2022 | Systematically develop and psychometrically evaluate a self‐report survey to measure patients' perceptions of participation in bedside handover | Australia | Cross-sectional | Hospital | Bedside handover | Health consumers | 4,6 |
| Tremblay et al. 2021 | A community-based participatory study to identify potential barriers and enablers to cultural safety in health care provided to Atikamekw living with diabetes in Québec | Canada | Qualitative | Clinic | Cultural safety | Organisational  and community partners | 2,4,6 |
| Troya et al. 2019 | Explores how older adults experience self-harm, identifying factors leading to self-harm | U.K | Qualitative | University and Community | Self-harm | Older adults as Patient and Public Involvement and Engagement group | 6 |
| Tyler et al. 2021 | Co-develop priorities for future quality and safety research projects and to develop a patient and public involvement and engagement group/steering committee for future research; and to raise awareness of quality and safety research and enable participants to consider their experiences in terms of quality and safety | U.K | Qualitative | Community | Patient safety | Patients | 4,6 |
| Tyler et al. 2023 | Develop a resident/carer measure of safety that can be used as a basis for proactively preventing and improving safety in care homes | U.K | Delphi method | Care homes | Adverse events | Patient and public contributors, residents, and carers | 4,6 |
| Van den Bulck et al. 2020 | Develop a set of electronic medical record-extractable and evidence-based quality indicators using a multidisciplinary expert panel, including patients, which can be used as a framework to evaluate and improve the quality of primary care for patients with chronic kidney disease | Belgium | RAND-modified Delphi method | Primary care | Patient safety | Patients | 4,6 |
| Van Strien‐Knippenberg et al. 2022 | Describe the process of information development about adjuvant breast cancer treatment, using cocreation with patients; and the key findings that emerged during this process | The Netherlands | Qualitative | Hospitals | Medication safety | Patients and patient advisor organisation | 4 |
| Wilson et al. 2021 | To understand the patients' knowledge of identifying information for their implanted devices and perspectives on sharing their implanted device information | USA | Qualitative | Hospitals | Communication | Patient research partners | 4 |
| Winterberg et al. 2022 | Describe how a human centered design model was used to develop an anxiety reduction app that can be seamlessly integrated into the fast-paced perioperative workflow during key stress points | USA | Iterative design research | Children’s Medical Center | Patient safety | Families | 4 |
| Yang et al. 2020 | Develop a Fear of Older Adult Falling Questionnaire-Caregivers and determine the content validity judged by content experts and caregivers; and determine the psychometric properties of the questionnaire and compare it with the parent Fear of Falling Questionnaire Revised | USA | Mixed methods | Primary care | Fall prevention | Caregivers | 4 |
| Young et al. 2018 | Co-design with patients and caregivers a tool for the development of managed access programmes | Canada | Participatory action research | Community | Medication safety | Patients and caregivers | 4 |
| Yuen et al. 2023 | Evaluate the feasibility and efficacy of a digital App developed to enhance patient communication with nurses during bedside nursing handover | Australia | Simulation study | Hospitals | Bedside handover | Patient actors/volunteers and patient representatives | 4 |
| Francis-coad et al. 2023 | Create a revised version of the resources for the Safe Recovery falls prevention education programme with staff and patients, and evaluate their reactions to, and learning from, the revised version of the Safe  Recovery programme | Australia | Exploratory sequential mixed method | Hospital | Fall prevention | Patients | 4 |
| Jo & Nabatchi 2019 | Investigate whether lay actors in a collective coproduction process experienced individual-level changes in: issue, awareness, perceived empowerment, trust in service professionals, and support for coproduction | USA | Randomised controlled experimental | Community | Diagnostic errors | Citizens as lay actors | 3,4 |
| O'Hara et al. 2018 | Explore what concerns about safety do hospital patients report; how do patients make sense of and categorise these safety concerns; and what is the incidence and nature of patient safety incidents experienced by patients | U.K | Randomised  controlled trial | Hospitals | Patient safety | Patient volunteers panel | 4 |
| de Jong et al. 2019 | Obtain a broad community perspective on three prototype audio-visual falls prevention messages using a community World Café forum approach | Australia | Mixed methods | Community | Fall prevention | Community dwelling older people | 3,4 |
| O'Donnell et al. 2019 | A co-design team was established at the commencement of the Systematic Approach to improving care for Frail older patients’ study to design and develop the frailty pathways for older people | Ireland | Quality improvement project | Hospital and community | Adverse events | Older people and advocates from community-based patient and public advocacy  organisations | 4 |
| Russ et al. 2020 | Assess the views relating to MySurgery with a cohort of diverse surgical patients recruited from the community and to understand perceptions of the app, perceived impacts on care and safety, and areas for improvement; and describe and evaluate the approach and impact of incorporating diverse patient and public involvement into the project design, planning, and delivery | U.K | Mixed methods | Community | Surgical safety | Public representatives | 3,4,6 |
| Mazuz et al. 2020 | Develop and validate a self-management tool of fall events which enables self-reporting and self-implementation of preventive interventions in the form of an online App called “Age-Techcare.” | Israel | Mixed methods | Community | Fall prevention | Community dwelling older people | 4 |
| Hjelmfors et al. 2018 | Describe the development of an intervention that is developed to improve communication about the Heart Failure trajectory and end-of-life care. Also, present data that provides a first insight in specific areas of feasibility of the intervention | USA | Pre and post interventional | Clinic | Communication | Patients and family members | 4 |
| Troya et al. 2019 | Critically reflect on the process, potential impact and identify challenges/ opportunities in involving robust patient and public engagement in doctoral research, including a systematic review and qualitative study | U.K | Systematic review and qualitative | University and Community | Self-harm | Older adults and carers as patient and public involvement group | 1,2,4,6 |
| Horgan et al. 2023 | Assess the impact of a complex quality improvement intervention on the incidence of surgical site (SSI) infection in patients undergoing elective colorectal surgery in an institution where SSI surveillance was not previously routinely undertaken | Ireland | Pre-test post-test design | Hospital | Surgical site infection | Patients | 4 |
| Tyler et al. 2020 | Obtain international consensus on a set of core outcome measures to be reported in all interventions intended to improve discharge from mental health inpatient services | U.K | Cross-sectional | Community | Discharge communication | Service users, families and carers, and patient and public involvement group | 4,6 |
| Ward et al. 2018 | Outline in detail the co-design processes employed in developing a collective leadership intervention for healthcare teams to improve team performance and patient safety culture | Ireland | Qualitative | Hospitals and community | Safety culture | Patient representatives and patient advocates | 4 |
| Berthelsen et al. 2023 | Develop an understanding of the concept of safety (harms) experienced by patients involved in clinical trials for their rheumatologic or musculoskeletal disease and seek input from the Outcome Measures in Rheumatology community before moving forward to developing or selecting an outcome measurement instrument | USA, Canada, Australia, and Europe | Qualitative | Community | Medication safety | Patients | 4 |
| Okkenhaug et al. 2023 | Validate a research tool for assuring quality in psychiatric health care by involving service users and health professionals, and explore the differences and points of similarity between the issues raised by service users and health professionals about the experience and risk of adverse events in psychiatric care | Norway | Qualitative | Hospital | Adverse events | Service users | 4 |

1 as joint grant holders or co-applicants on a research project; 2 identifying research priorities; 3 as members of a project advisory or steering group; 4 commenting on and developing research materials; 5 data collection (undertaking interviews with research participants); 6 carrying out research as user and/or carer researchers.
